# G protein gamma subunit, a hidden master regulator of GPCR signaling

**DOI:** 10.1016/j.jbc.2022.102618

**Published:** 2022-10-19

**Authors:** Dinesh Kankanamge, Mithila Tennakoon, Ajith Karunarathne, N. Gautam

**Affiliations:** 1 Department of Anesthesiology, Washington University School of Medicine, St Louis, Missouri, USA; 2 Department of Chemistry, St Louis University, St Louis, Missouri, USA; 3 Department of Genetics, Washington University School of Medicine, St Louis, Missouri, USA

**Keywords:** GPCR, γ subunit, signaling, translocation, optogenetics, cancer, ER, Endoplasmic Reticulum, GPCRs, G protein coupled receptors, GTP, Guanosine-5′-triphosphate, PIP3, Phosphatidylinositol (3,4,5) trisphosphate, PI3Kγ, Phosphoinositide 3-kinase γ, PLC, Phospholipase C

## Abstract

Heterotrimeric G proteins (αβγ subunits) that are activated by G protein-coupled receptors (GPCRs) mediate the biological responses of eukaryotic cells to extracellular signals. The α subunits and the tightly bound βγ subunit complex of G proteins have been extensively studied and shown to control the activity of effector molecules. In contrast, the potential roles of the large family of γ subunits have been less studied. In this review, we focus on present knowledge about these proteins. Induced loss of individual γ subunit types in animal and plant models result in strikingly distinct phenotypes indicating that γ subtypes play important and specific roles. Consistent with these findings, downregulation or upregulation of particular γ subunit types result in various types of cancers. Clues about the mechanistic basis of γ subunit function have emerged from imaging the dynamic behavior of G protein subunits in living cells. This shows that in the basal state, G proteins are not constrained to the plasma membrane but shuttle between membranes and on receptor activation βγ complexes translocate reversibly to internal membranes. The translocation kinetics of βγ complexes varies widely and is determined by the membrane affinity of the associated γ subtype. On translocating, some βγ complexes act on effectors in internal membranes. The variation in translocation kinetics determines differential sensitivity and adaptation of cells to external signals. Membrane affinity of γ subunits is thus a parsimonious and elegant mechanism that controls information flow to internal cell membranes while modulating signaling responses.

G protein-coupled receptors (GPCRs) on the plasma membrane sense external signals and activate heterotrimeric (αβγ) G proteins. Activation of the G proteins results in the α subunit exchanging GDP for GTP and the dissociation of a tightly associated βγ complex. Both the α-GTP and the βγ complex are independently capable of modulating the activity of effectors. The α subunits of G proteins are GTPase switches that are active in the GTP-bound form and deactivated in the GDP bound form (1). α and βγ subunits are large families of diverse members that act on a number of effectors such as adenylyl cyclase and phospholipase C (2, 3). The βγ complex acts on various effectors including G protein-gated inwardly rectifying K^+^ channels (4), adenylyl cyclase (5), phospholipase C (PLC) (6), GPCR kinases (7), and phosphoinositide 3-kinase γ (PI3Kγ) (8).

Recent reviews have focused on various aspects of the α and βγ subunits (9, 10, 11). In contrast, studies of the γ subunits have been limited and their structure and potential functions have not been reviewed. This review focuses on present knowledge about the γ subunits, their potential roles in signaling based on this information, gaps that remain in our knowledge, and potential future experimental directions that can address these lacunae.

## A history of G protein γ subunits

The γ subunit of transducin, the G protein found in rod outer segments of the retina was the first γ to be characterized at the protein and cDNA level (12). The identification of the cDNA for a γ subunit associated with the Gi/o proteins using peptide analysis and PCR showed that the primary structures of the two γ subunits diverged considerably, and it was evolutionarily related to the small GTP binding Ras family of proteins (13). Identification of additional subunits suggested that the γ subunits were potentially a large family of structurally diverse proteins (14). Over the years, 12 γ subunit types were identified based on cDNA sequences (14, 15, 16, 17, 18, 19, 20, 21). The primary structures of the γ subunits were conserved in different mammalian species indicating that the differences in amino acid residues among these subunits were of functional importance (3). The presence of a γ subunit in yeast (22) and γ subunits in plants (23, 24) also showed that the G protein γ subunit has been retained over a long period of evolution in all eukaryotes and further emphasized the potential for an independent role in signaling. In the plant *Arabidopsis thaliana*, an atypical γ subunit has been identified with a primary structure that is distinctly different from all other γ subunits (25). This suggests that the γ subunits have evolutionarily diverged considerably in plants to play specialized roles.

Though the lipidation of α subunits with a covalent 16-carbon palmitate group and/or 14 carbon myristate group at their N terminus was discovered in the late 1980s (26), it was only in 1990 that the anchoring of βγ to the membrane *via* a prenyl moiety by covalent posttranslational modifications at the C terminus of γ subunit was identified (27, 28, 29). γ subunits are lipidated with a prenyl group, either farnesyl (15 Carbon) (27, 30) or geranylgeranyl (20 Carbon) (31, 32), through a stable thioether linkage to the C-terminal Cys. A four-residue conserved amino acid sequence called; “the CaaX motif” on the C terminus of the γ subunit determines the type of prenylation on a specific γ subtype. CaaX is composed of a Cys, two aliphatic amino acids-aa, and a prenyl transferase determining residue, X. The Cys is farnesylated when X is Met, Ser, Glu, or Ala (as in γ1, γ9, and γ11), and geranylgeranylated when X is a Leu (the rest of the nine γ subunits) (33). The last three residues (aaX) of prenylated γ are proteolytically cleaved off by an endoprotease; Ras converting CaaX endopeptidase, and subsequently the prenyl Cys is carboxy methylated by a methyltransferase, isoprenyl-cysteine carboxyl methyl transferase (26, 34).

In contrast to prenylation which is restricted to a small set of proteins and is retained through the life of the modified proteins, phosphorylation is ubiquitous and transient. Phosphorylation of γ subunits was shown to occur in the case of γ12, and the results suggested a role for the phosphorylation in effector regulation in specific G protein pathways (18, 35, 36). More recently, phosphorylation of the yeast γ subunit has been shown to be essential for downstream signaling activity (37). An examination of the sequences of γ subunits has shown that eight of the subunits contain putative phosphorylation sites in the N-terminal 14 residues (38). In the future it will become clearer whether these sites are phosphorylated, and it is a general theme in regulating the activity of these subunits.

Since there is a possibility that βγ complexes made up of different combinations of β and γ subunit types could have distinct functions, it was important to determine the rules for the association of various β and γ subunit types. Do all β subunits associate with all γ subunits or is there a selective association? Such selectivity would suggest that even if a cell expresses many subunit types, only certain βγ complexes are possible. A variety of experimental methods showed that associations between β and γ subunit types were selective (39, 40, 41, 42). Importantly, purifying native βγ complexes from tissues has confirmed selective association between β and γ subtypes (43).

When individual G protein heterotrimers based on α subunit identity from different tissues were examined, they were found to contain different γ subunits suggesting again that the γ subunit types play different roles (14, 44, 45, 46, 47). Knocking down individual γ subtypes in a cell line with antisense oligonucleotides provided support for such specific roles by selectively affecting distinct signaling pathways (48).

After the early mapping of the mouse genes and the elucidation of the structure of a γ subunit gene (49), the genomics of γ subunits is now comprehensive in both mouse and human (Tables 1 and 2). The earlier studies showed that genes for two subunits γ1 and γ11 which are closely related by homology are arranged together in a head to tail orientation suggesting that they may have arisen as a result of gene duplication, and the γ3 gene is also in a head to tail orientation with a gene *Gng3lg* (20). This gene was later named in humans as *BSCL2*, and mutations in this gene are associated with congenital lipodystrophy, Berardinelli–Seip syndrome (50).

**Table 1:** Mouse γ subunit genes^a^

Gene symbol	Gene id	Chromosome no	Number of exons
GNG1	14,699	6	6
GNG2	14,702	14	8
GNG3	14,704	19	3
GNG4	14,706	13	5
GNG5	14,707	3	3
GNG7	14,708	10	7
GNG8	14,709	7	6
GNG9	14,710	11	7
GNG10	14,700	4	3
GNG11	66,066	6	2
GNG12	14,701	6	6
GNG13	64,337	17	4

a Data adapted from database resources of the National Center for Biotechnology Information.

**Table 2:** Human Gγ subunit genes^a^

Gene symbol	Gene id	Chromosome no	Number of exons
GNG1	2792	7	3
GNG2	54,331	14	14
GNG3	2785	11	5
GNG4	2786	1	8
GNG5	2787	1	4
GNG7	2788	19	6
GNG8	94,235	19	5
GNG9	2793	17	5
GNG10	2790	9	3
GNG11	2791	7	2
GNG12	55,970	1	7
GNG13	51,764	16	3

a Data adapted from database resources of the National Center for Biotechnology Information.

There were suggestions that the specific role in signaling that γ subunit types play is through selective and direct interaction with receptors. Studies with purified proteins showed that the βγ complex was an obligatory requirement for receptor activation of the α subunit (1, 51). A set of results suggested that the γ subunit interaction with a receptor is a requirement for G protein activation. Peptides from the C-terminal domain of the γ1 subunit stabilized the photoactivated form of rhodopsin, and mutations in this region prevented heterotrimer activation by rhodopsin (52, 53). Consistent with these results, a conformational change in the C-terminal domain peptide of γ subunit when bound to light-activated rhodopsin was detected while the same peptide remained disordered in the presence of inactive dark-adapted rhodopsin (54). A geranylgeranylated peptide corresponding to the C terminus of γ5 subunit, but not γ7 or γ12 subunits were shown to inhibit M2 muscarinic receptor signaling, also indicating Gγ-receptor interactions (55). This role for the γ subunit is also supported by findings that particular γ subunit types are more potent in supporting G protein activation by a receptor (47, 55, 56, 57, 58).

There are 20 available structures of the receptor–G protein heterotrimer, all of them containing γ2 with or without the prenylation site. Their PDB IDs and the particular receptor–G protein complex (Table 3). The structure of the complete C-terminal domain of the γ subunit is not clear in any of these structures likely due to the hypervariable nature of the C-terminal domain. Since structures of the GPCR-G protein complex capture frozen states of this dynamic interaction in a narrow time window, it is possible that they have not captured the states when direct interaction between the receptor and the γ subunit occurs. Consistent with this notion, recent modeling shows how the existent findings fit into a model of receptor–G protein interaction where the γ subunit tail interaction occurs transiently with an intracellular hydrophobic site in the receptor facilitating subsequent interaction with the α subunit (59). Structures in the future that capture transient states of the receptor–G protein complex after activation can more directly address questions about the interaction of the γ subunit with the receptor.

**Table 3:** Cryo-EM and X-ray crystallographic structure information of different GPCR-G protein complexes^a^

a Structural data of different GPCR-G proteins adapted from Protein Data Bank.

## Expression of individual γ subunits in tissues

Once it was determined that γ subunits are a family, their expression in mammalian tissues was examined (Table 4). There were early suggestions that γ subtypes are expressed selectively in mammalian tissue. When antisera specific to γ2 and γ3 subunits were used, they were detected in G protein heterotrimers purified from brain but not some other tissues (14). The presence of γ2 and γ3 was further established when brain extracts were examined for G protein γ subunits. These studies showed that γ5, γ10, and γ11 were present in several different tissues, although γ5 and γ10 were barely detectable in brain (60, 61, 62). This selectivity in mammalian tissues was an indication that they have distinct roles.

**Table 4:** Genomic location and tissue-specific expression of human Gγ subunits^a^^,^^b^

Gene symbol	Gene id	Chromosome no	Number of exons	Tissue-specific expression (protein/RNA^c^)
GNG1	2792	7	3	Retina
GNG2	54,331	14	14	Brain and smooth muscles
GNG3	2785	11	5	Brain^c^
GNG4	2786	1	8	Brain and endocrine tissues^c^
GNG5	2787	1	4	Ubiquitous
GNG7	2788	19	6	Brain
GNG8	94,235	19	5	Brain^c^
GNG9	2793	17	5	Retina
GNG10	2790	9	3	Ubiquitous^c^
GNG11	2791	7	2	Endocrine, liver, and muscle tissues
GNG12	55,970	1	7	Placenta, fat tissues, and bronchus
GNG13	51,764	16	3	Brain and retina

a Genomic data adapted from database resources of the National Center for Biotechnology Information.

b Tissue-specific expression data adapted from database resources of the Human Protein Atlas.

c RNA expression data.

In a few specialized cell types, only one γ subunit type is predominantly expressed. In rod photoreceptors of the mammalian retina, γ1 is the subunit type that is mainly detectable (63). Similarly in cone photoreceptors that play a role in color vision, γ9 is expressed (64). Taste receptor cells contain γ13 (21).

More recently, a large-scale project to localize proteins in human tissues confirms that the G protein γ subunit types are expressed differentially in various tissues (65). These results from immunohistochemistry confirm earlier findings that γ1 and γ9 are expressed at high levels in rod and cone photoreceptors and not detected in other tissues; γ2 is widely expressed but highest in brain and smooth muscle; γ5 is broadly ubiquitous; γ7 is restricted in its expression to the brain; γ12 is expressed at high levels in ciliated and glandular cells; γ13 was not examined in taste receptor cells, but it was detected at high levels in cerebellar Purkinje cells, endocrine cells of the gastro intestinal tract, and in the inner part of the retina. The inner retinal expression confirms an earlier finding that γ13 is expressed in bipolar cells of the retina (66). γ11 which is closely related to γ1 and γ9 in primary structure and is similarly farnesylated was not expressed at high levels in any of the tissues examined. It is predominantly detected in glandular cells. Based on the strong similarities in the properties of this subunit type with γ1 and γ9 that are described in the latter part of this review, it is possible that it is expressed at high levels in a specialized cell type that has not yet been examined. Although γ8 was not examined in this analysis, previous findings showed that it is expressed in olfactory and vomeronasal neurons of mice (19). Although it was absent in whole brain RNA, a detailed study of the expression of γ subunits in different parts of the rat brain detected γ8 RNA at high levels in the habenula (67). As mentioned in the case of γ11 and γ13 above, this emphasizes the need for examining individual cell types to determine the actual expression patterns of the γ subtypes since they maybe expressed in cell types that may be a relatively small proportion of a particular tissue.

Going forward it will be valuable to identify the predominant β and γ subtypes in a cell type more clearly. Given the selectivity in associations between β and γ subunit types, this will help determine how each of these βγ complexes modulate the signaling activity in a particular cell.

## Effects of disrupting the expression of individual γ subunits

Many of genes for the γ subunits have been specifically ablated in mice. Knocking out γ subunits results in dramatic phenotypic deficits in the animals. *GNGT1* gene knockout results in the absence of γ1 subunit in rod photoreceptors and leads to their progressive degeneration (68). *GNG3* knockout animals have low body weight and are susceptible to seizures (69) while the knockout of γ7 subunit which is also expressed in the brain has distinctly different effect resulting in a muted response to caffeine (70). In each of these knockout mice, there is also a significant reduction in the levels of the α and β subunit specific to the cell types expressing these γ subunit types, suggesting that γ subunits are required for the stability of the α and β subunits. The γ2 subunit has been knocked down by treating mouse brain with antisense oligonucleotides (71, 72). These mice demonstrate a significantly reduced nociceptive response to opioid, cannabinoid, and adrenoreceptor types. Knocking out *GNG5* lead to embryonic lethality with cardiac defects (73) and with the disruption of γ8 expression, the knockout mice demonstrated learning and memory defects consistent with the expression of γ8 in the habenular region (74). In addition, consistent with the expression of this subunit type in the vomeronasal neurons *GNG8* knockout mice were found to be defective for their response to pheromones and showed a consequent decrease in aggressive behavior (75).

Similar to γ8, the γ13 subunit is expressed in different tissues—the olfactory neurons as well as in the retinal bipolar cells. Knocking out the *GNG8* gene selectively in the olfactory tissue alone resulted in mice that showed a poor olfactory response (75). When *GNG13* was knocked out in all tissues and the mice were examined for any effects of the absence of γ13 in the bipolar cells of the retina, they were found to be defective in their light response (76).

These specific effects of the loss of particular γ subunit types that are seen in an animal system have also been found in plants. The diversity of γ subunit types seen in animals is reflected in plants. Diploid plant species such as rice and Arabidopsis contain only one α and β subunit type but express several γ subunit types. Altered expression of γ subunit types has profound effects on various phenotypes of plants (77, 78).

The results of inducing the loss of individual γ subunit types on animal and plant systems has shown convincingly that the G protein γ subunits play important but distinctly different roles in governing the normal development and function of various cell types in animals and plants.

## G protein γ subunits in cancer

Consistent with the effect of knockdowns and knockouts of γ subunit types showing specific and significant defects in mice, altered expression of γ subunit types has been shown to be associated with disease, mainly cancer.

Several human malignant melanoma cell lines expressed low levels of γ2 compared to normal melanocytes (79). Knocking down γ2 expression in the parental cell line enhanced migration and invasiveness and increased focal adhesion kinase activity. Overexpressing γ2 in melanoma cells reduced migration and invasion of melanoma cells as well as focal adhesion kinase activity. These findings suggest that γ2 downregulation was at the basis of the metastatic properties of these cells.

Glioblastomas were found to contain downregulated γ4 due to high levels of methylation (80). Glioblastoma cell lines similarly contained downregulated γ4. When γ4 was expressed in these cells, cell proliferation was inhibited. γ4 expression also inhibited Ras-induced transformation of astrocytes and CXCR4-induced activation of downstream effector kinases. Since CXCR4 upregulation is known to play a role in glioblastoma proliferation and motility, GNG4 appears to act as a tumor suppressor in these cells. This was consistent with an earlier finding that expressing γ4 in a renal carcinoma line reduced proliferation (81).

Similar association between downregulation of a γ subunit and association with pathological cell proliferation has been shown in the case of the γ7 subunit. In the majority of esophageal cancer tissues examined, γ7 expression was low (82). There was an association between hypermethylation and reduced expression of γ7. In an earlier study from the same group, growth of cell lines originating from various gastrointestinal cancers was inhibited by overexpression of γ7. In a nude mouse model, there was inhibition of the growth of tumor cells into which γ7 had been introduced (83). In a more recent study, in a third of the tumors of the larynx and floor of the mouth that were examined, γ7 subunit expression was absent (84). Close to half the tumors also showed hypermethylation of the γ7 subunit gene. Although not ubiquitous, the hypermethylation seen in the case of the γ7 gene in these reports is reminiscent of the hypermethylation of γ4 in glioblastomas mentioned earlier.

While these findings suggest that γ subunits can act as tumor suppressors, in other cases γ subunit types appear to act as tumor promoters. High levels of γ4 subunit expression were found in primary gastric cancer cells and in cells that had metastasized to the liver (85). In a mouse liver metastasis model system, there was a significant reduction in tumor formation by cells in which γ4 was knocked out. Similarly, the γ9 subunit was expressed at high levels in prostate cancer cell lines compared to their expression in cells from which these lines originated (86). When the γ9 subunit was knocked out in a prostate cancer cell line, there was significant reduction in the ability of these cells to migrate and invade suggesting that the γ9 subunit played a role in metastasis (87).

The ability of the γ4 subunit to act as a tumor suppressor in some cancers and as a tumor promoter in others is intriguing. It is possible that the roles of different γ subunits are singularly dependent on the internal molecular milieu of individual cell types. The specific GPCR, α subunit, and effectors present in a particular cell type may define the impact of the activity of the βγ complex containing a specific subunit type.

These reports above that provide evidence for a role of different γ subtypes in pathological cell proliferation and metastasis in disparate tissue types suggest that G protein γ subunit misregulation can underlie cancer.

Overall, these results as well as those described in the earlier section of the striking effects of loss of γ subunit type expression in whole animal or plant systems do not directly provide clues about the mechanistic basis of these effects. Future studies will need to mechanistically focus on the role of γ subunits in different cell types to understand why the loss of a subunit leads to striking phenotypic changes in a mouse or why the altered expression of a γ subunit leads to the diseases seen in a human. It will also become clearer over time whether γ subunit types can be targeted selectively to control cancer cell proliferation and metastasis and additionally if the misregulated γ subunit types can serve as cancer markers that can be used as prognostic tools.

The sections below focus on one unexpected molecular mechanism that may explain this obligatory requirement for the γ subunit for normal cell function and development. It also suggests a rationale for the specificity of effects described above resulting from the loss of a γ subunit and its downregulation or upregulation.

## Receptor-activated translocation of the G protein βγ complex

**Figure 1:**
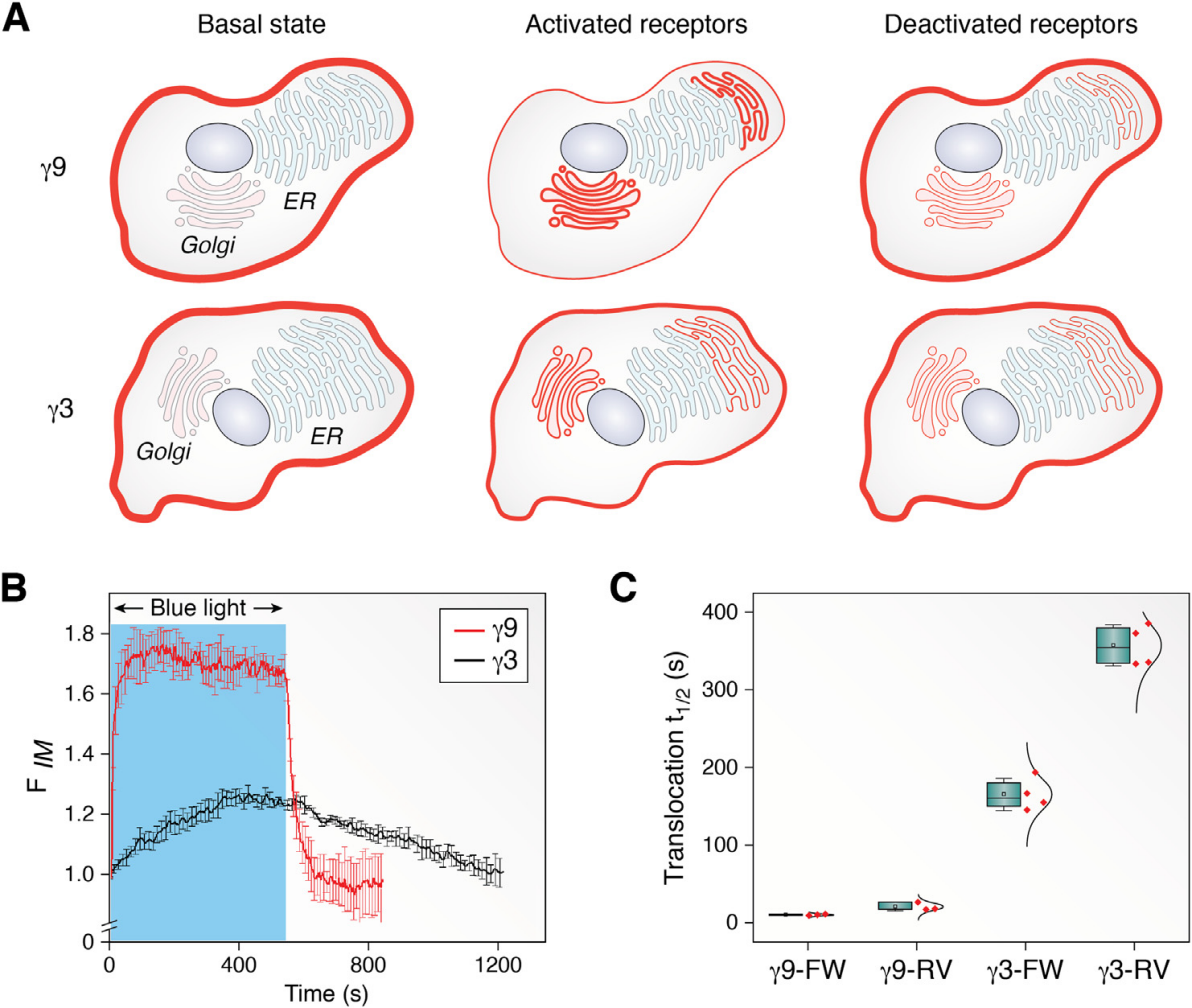
**βγ complex translocation in cells.***A*, representative cartoon based on images of fluorescent protein tagged βγ9 (fast translocating) and βγ3 (slow translocating) complexes in cells showing distinct forward and reverse translocation magnitudes upon receptor activation and deactivation. *B*, traces quantitating the fluorescence intensity at internal membranes (ER and Golgi) show the rates and magnitudes of forward and reverse translocation of βγ9, and βγ3 complexes in images of cells expressing a *blue* light–sensitive opsin GPCR. *Blue* light exposed duration is *shaded blue*. F_IM_: fluorescence at internal membranes normalized to basal fluorescence intensity. *C*, translocation half times (t_1/2_) calculated from traces in B show significant difference between βγ9 and βγ3 complexes. βγ9: 11s forward and 21s reverse. βγ3: 265s forward and 357s reverse (FW: Forward, RV: Reverse). GPCR, G protein-coupled receptor.

The early studies of G proteins predominantly relied on experiments with purified proteins or with lysed cells expressing appropriate cDNAs. While these methods provided valuable information about the function of these proteins, it was unclear how these proteins functioned in an intact live cell. The ability to tag G protein subunits with fluorescent proteins without altering their properties allowed their dynamic behavior to be observed in a live cell before and after receptor activation with or without specific pharmacological or genetic perturbation. This shift in the experimental paradigm to observing the behavior of G protein subunits in living cells in real time by capturing 3D images at high speed altered the view of heterotrimeric G proteins as molecules that function at the inner surface of the plasma membrane. When the α, β, and γ subunits were individually observed in a living cell in the basal state, they were found to be constantly moving back and forth between the plasma membrane and internal membranes (88). When receptors were activated, the G protein βγ complex translocated away from the plasma membrane to intracellular membranes (89). βγ complexes with different γ subunits translocated at different rates and were targeted to different intracellular membranes—Golgi or ER (Fig. 1) (90, 91, 92). Various receptors and α subunit types supported the translocation, suggesting that it is a conserved process (93).

**Figure 2:**
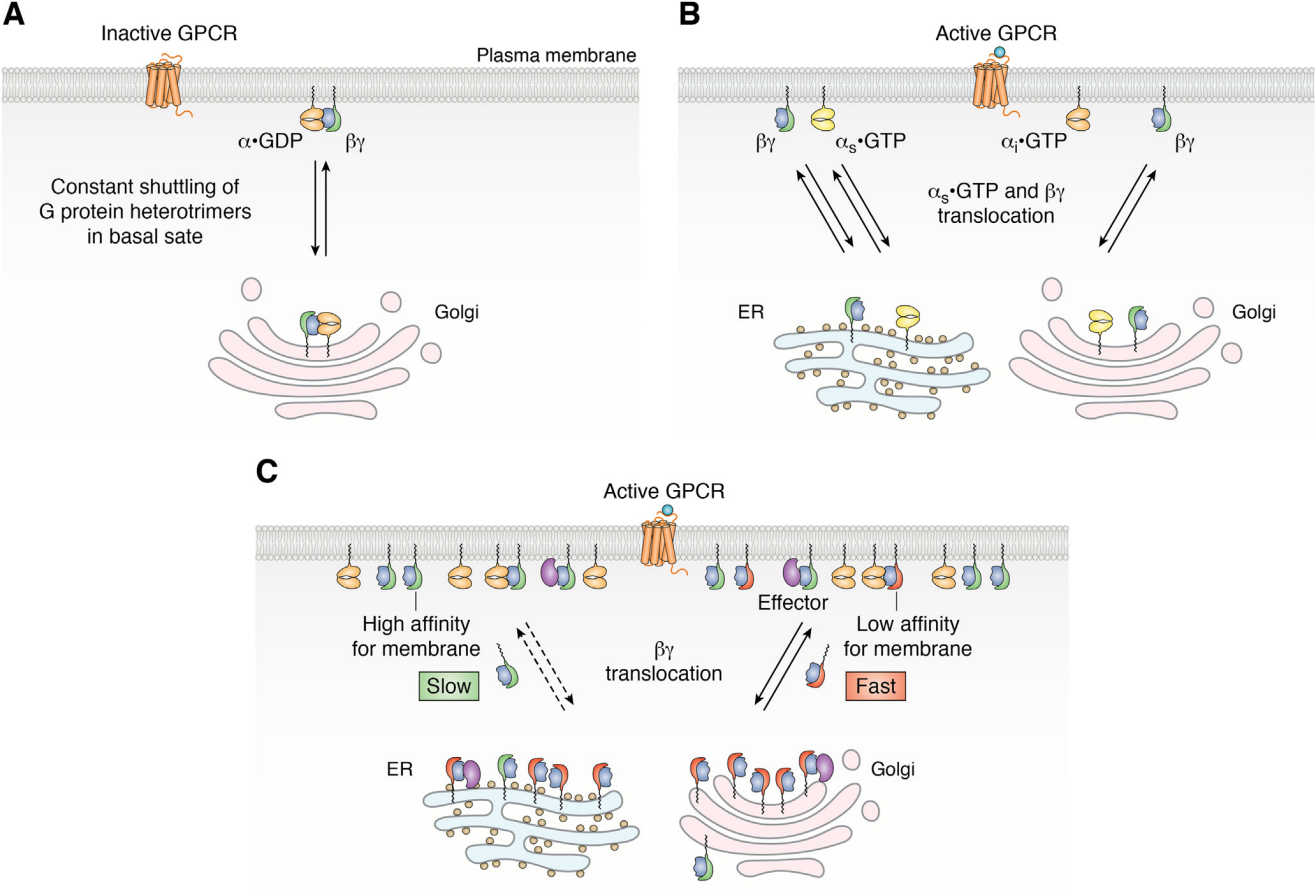
**Dynamics of G protein subunits.** Cartoon representation of how G protein heterotrimers and their subunits are capable of movement within cells in the basal state and after receptor activation based on images of cells containing fluorescent protein-tagged subunits. Numbers of representative molecules shown do not reflect the actual stoichiometry in living cells. *A*, G-protein heterotrimers are constantly shuttling between plasma membrane and internal membranes in the basal state. *B*, αs subunit transits into the cell on activation unlike other α subunit types. *C*, βγ complexes containing different γ subunits translocate at different rates to internal membranes on receptor activation. βγ complexes containing γ subunits with high affinity for the membrane (*green*) translocate at a slow rate while those containing γ subunits with low affinity (*red*) translocate fast. As a result, after receptor activation the number of slower βγ complexes (*green*) is higher on the plasma membrane compared to the internal membranes while with fast translocating βγ complex (*red*), it is higher in the internal membranes compared to the plasma membrane. This allows slower βγ complex to activate effectors (*purple*) at the plasma membrane more effectively than the fast βγ complex. GPCR, G protein-coupled receptor.

G proteins associate with membranes due to lipid modifications—myristoyl and/or palmitoyl on the α subunit and prenyl on the γ subunit (11). The shuttling of the G protein heterotrimer between the plasma membrane and internal membranes suggested that there was dynamic loss and re-modification of a lipid. 2-bromopalmitate inhibition of shuttling suggested that it is likely the result of a palmitoylation cycle (88). This was established with the αq subunit which was shown to undergo a palmitoylation-depalmitoylation cycle and the enzyme at the basis of the cycle was identified (94). In contrast to this shuttling seen of α subunits in the basal heterotrimer state, the translocation of αs-GTP was found to occur after receptor activation (95) (Fig. 2, *A* and *B*).

**Figure 3:**
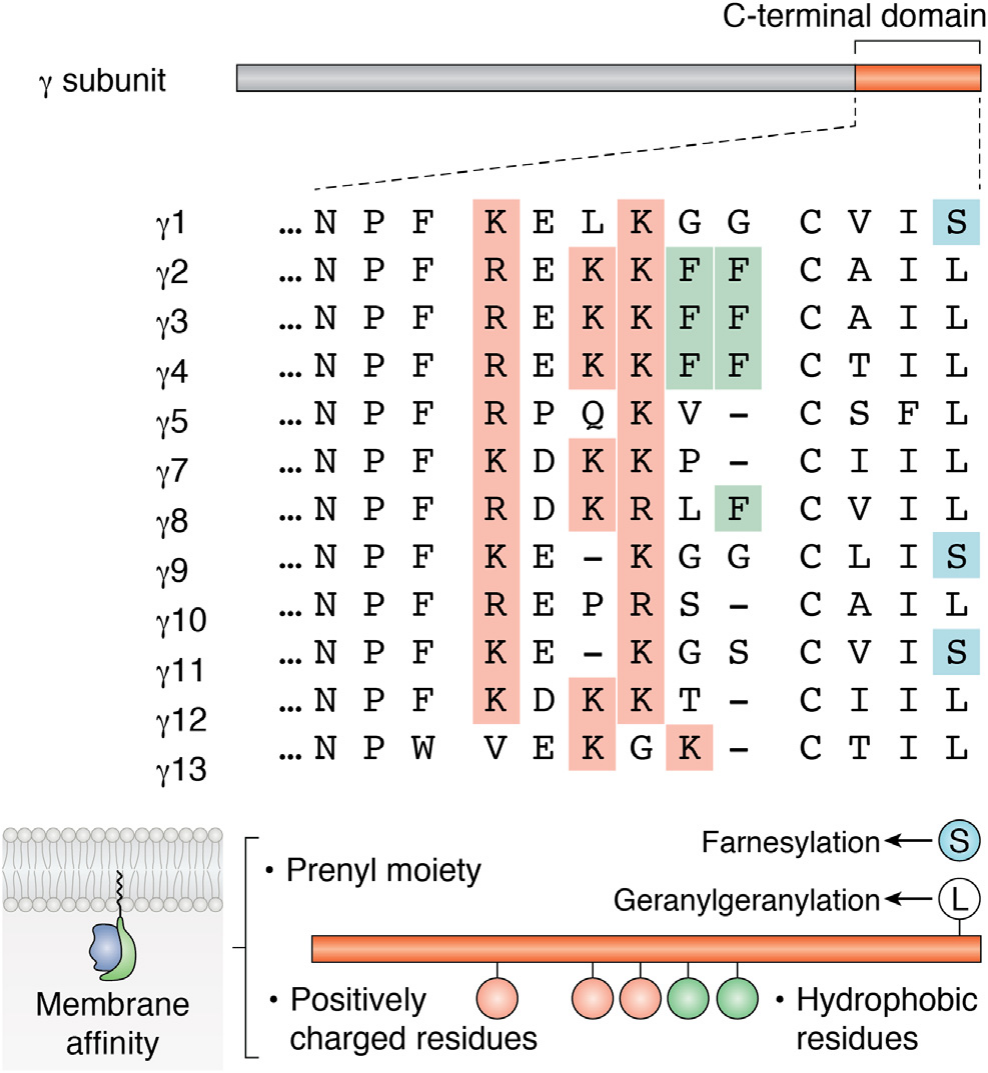
**Amino acid sequences of the C-terminal domain of γ subunits.** C-terminal domain of the γ subunit starts from a conserved NPF amino acid sequence. The sequence alignment was performed using the MUSCLE alignment tool at EMBL-EBI (https://www.ebi.ac.uk/Tools/msa/muscle/). γ subunits with a C-terminal S (ser, *blue*) are farnesylated. While those with L (leu, *white*) are geranylgeranylated. Membrane affinity is determined by the type of prenyl moiety, as well as the number of hydrophobic (*green*) and positively charged residues (*pink*) in a particular γ subunit.

The shorter 15-carbon farnesyl lipid has lower hydrophobicity and thus affinity for membranes compared to the 20-carbon geranylgeranyl moiety. Live cell imaging of C-terminal mutants of γ subunits, measuring the dissociation of prenylated fluorescent peptides corresponding to the γ subunit C terminus and mathematical modeling showed that apart from the prenyl moieties, variations in a set of hydrophobic and basic residues at the C terminus of the γ subunits determine differential membrane affinity among γ subunits and consequently translocation properties (Fig. 3) (92). The electrostatic interactions between positively charged residues and polar headgroups of membrane phospholipids in this region enhances affinity. Further analysis of the role of residues at the C-terminal region immediately upstream of the prenyl group has established that translocation rate differences are determined by alterations of a LysLysPhePhe sequence conserved in the γ2, 3, and 4 subunits (Fig. 3) (92, 96, 97).

A phylogenetic tree of the C-terminal domain residues starting from a conserved AsnPro in the γ subunits shows that 12 γ subunits can be classified into three different subgroups based on the physicochemical properties of amino acids in this sequence. The sequence properties are reflected in the translocation behavior of the γ subunits that are part of a βγ complex as seen in their grouping based on translocation rates (Table 5).

**Table 5:** Primary structures of γ subunit C terminus determines translocation properties

	γ subtype		Translocation efficacy (Translocation T_1/2_)	Membrane affinity (% loss from PM)	Type of lipid attached
	γ^2^		Slow (181–270 s) (97)	High (26–30%)	Geranylgeranyl
	γ^3^				
	γ^4^		Medium (41–124 s) (97)	Medium (35–51%)	Geranylgeranyl
	γ^13^				
	γ^5^				
	γ^10^				
	γ^8^				
	γ^7^				
	γ^12^				
	γ^11^		High (5–38 s) (97)	Low (67–80%)	Farnesyl
	γ^1^				
	γ^9^				

Why do translocated subunits accumulate in internal membranes? Do they translocate to specific organelles? How does a lipidated protein complex traverse through the cytosol?

It has been shown using different methods that the shuttling of the heterotrimeric G protein between the plasma membrane and internal membranes is likely diffusive (88). Translocation of the βγ complex also occurs through diffusion and not through vesicle trafficking (98). Thus, the mode of transit across the cytosol is unlikely to contribute to the translocation kinetic differences among different βγ subunit complexes. This is consistent with evidence mentioned above that membrane affinity of the γ subunit type is the primary determinant of βγ complex translocation rate differences.

**Figure 4:**
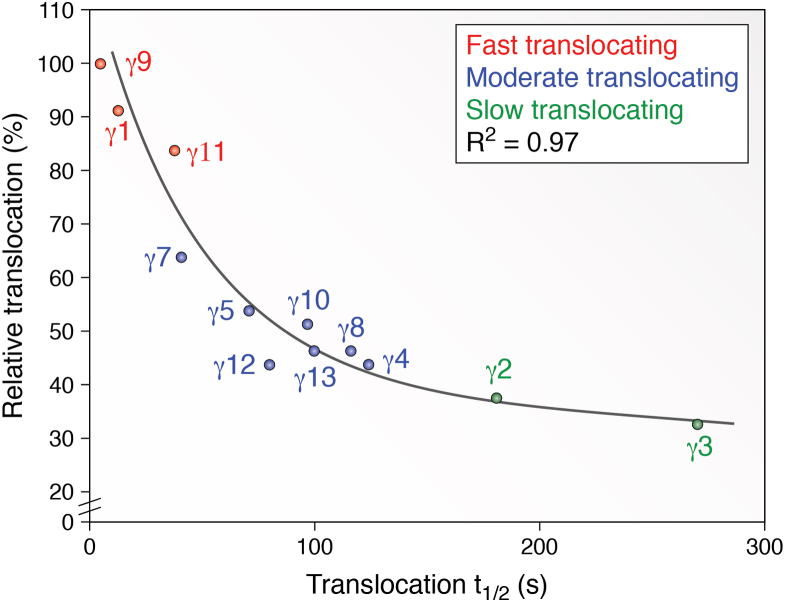
**Relative percent translocation magnitudes of γ family members normalized to γ9 at internal membranes *versus* their corresponding translocation kinetics.** The relative percent translocation internally was adapted from Senarath *et al* (96). The magnitude of translocated γ subunit was determined as a percent increase over basal level fluorescence intensity in internal membranes and normalized to that of the γ9 subunit. Translocation was induced by the activation of *blue* opsin.

When a significant proportion of βγ is released rapidly from the plasma membrane as in the case of rapidly translocating subunits, it can explore the surfaces of internal membranes that have a 10- to 20-fold higher surface area compared to the plasma membrane. At steady state, even if affinities are similar for the plasma membrane and internal membranes, the internal will have bound a higher concentration of fast translocating βγ subunits than the plasma membrane (Fig. 2*C*). In the case of slow translocating βγ subunits, the proportion found in internal membranes would be less (Fig. 2*C*). The experimental behavior of the different βγ subtypes does reflect this simple model (Fig. 4).

There are some differences between the targeting of the βγ complex to different intracellular organelles dependent on the γ subtype. Most translocated βγ complexes predominantly translocate to the Golgi while γ13 translocates mainly to the ER. A recent report that all γ subunits translocate to multiple internal membranes has been performed with a high affinity bait targeted to these membranes which may bias the distribution toward such ubiquity (99).

Future studies to more clearly establish the target of translocating subunits need to be performed at higher resolution where membrane targeting is solely determined by the intrinsic properties of the membranes and γ subunit types in their native state.

A question that remains is how a lipid modified protein like the γ subunit is able to diffuse through the cytosol when translocation occurs. In a HeLa cell, the approximate distance from the plasma membrane to the Golgi is 20 μm. Based on calculations, a fluorescent protein–tagged βγ complex will take 9.5 s for the root mean square of this displacement (92). The experimentally determined half time for translocation of the βγ9 complex to the Golgi after receptor activation is about 9 s (92). This suggests that diffusion is the rate limiting factor and not the dissociation of subunits from membranes because the farnesylated peptide corresponding to the γ9 C-terminal domain dissociates from membranes with a t_1/2_ of milliseconds. It also indicates that any additional steps would have slowed down this translocation significantly compared to plain diffusion so the cytosolic transit of the βγ complex is unlikely to be aided by a binding protein that masks the prenyl moiety as in other GTP binding proteins like Ras, Rab, and Rho (100, 101). It is likely that the prenyl moiety is actually obscured from the hydrophilic environment as it passes through the cytosol within the βγ complex. There is some evidence that supports this possibility. The structure of the βγ complex has been shown to be in two states where in one state the prenyl moiety is located in a prenyl binding site inside the β subunit (102). For this question to be fully addressed, it will require the intact C-terminal fully processed tail to be structurally resolved in the fraction of the βγ complex that is in transit between membranes.

A cell often encounters stimuli localized to one portion of it. What is the effect of inducing βγ complex translocation in a part of a cell? G protein signaling at the cell surface is known to induce completely different effects compared to that at inner membranes. For instance, β-adrenergic receptor and G protein signaling at the cell surface is cardioprotective, while deeper membrane signaling causes cardiac hypertrophy and cardiomyocyte apoptosis (103, 104). At present this area has not been much explored. The wide variety of optogenetic tools that have become available along with methods to obtain high resolution 3D images at high speed can help in determining more accurately the specific subcellular distribution of translocated Gβγ complex and its impact on cell function in contrast to global activation.

## Functional basis of βγ complex translocation

Although the conventional view of GPCR-mediated G protein signaling was that it was restricted to the plasma membrane, more recent evidence suggests that G proteins are present and function at intracellular locations such as the ER, nucleus, and Golgi complex (9, 98, 105). However, the ability of a GPCR on the plasma membrane to induce the translocation of the βγ complex to various organelles introduces a distinctly different paradigm in signaling. It suggests that GPCRs can act at a distance on effectors at internal membranes and modulate their activity.

When the potential role of βγ complex translocation in internal membranes was examined, it was found that rapidly translocating βγ11 subunits were capable of inducing Golgi vesiculation (106). In contrast, a βγ3 complex that translocated slowly had no effect. Additionally, the rapidly translocating subunits enhanced insulin secretion in insulinoma cells. Though the βγ complex in a reconstitution assay has been shown to stimulate Golgi fragmentation *via* a PKD- and PLCβ-mediated pathway (107) and Golgi localized βγ complex can regulate protein transport from the trans-Golgi network to the cell surface (108) it had earlier been unclear how the βγ complex reached the Golgi. A follow-up study further demonstrated that γ11 subunit also regulates cellular senescence by acting on the Golgi structure in response to GPCR activation (109).

Similarly, subtype-dependent βγ complex translocation to the Golgi complex regulates the ERK pathway and cancer metastasis through PI3Kγ activation (87). Knockout of fast translocating γ9 subunit in human prostate cancer and HEK293 cells exhibited markedly reduced ERK1/2 activity in the Golgi, whereas knockdown of slow translocating γ3 subunit did not have a significant effect. Also, the knockdown of γ9 subunit and p110γ subunit of PI3Kγ strikingly inhibited the prostate cancer cell migration, invasion, and metastasis upon chemokine receptor activation. The same research group also showed that βγ translocation to the Golgi controls ARF1 activation through PI3Kγ (110). Both these studies show how βγ translocation can provide a mechanism for a GPCR to control signaling pathways at internal membranes.

The differences in the kinetics of βγ complex translocation is retained regardless of the receptor, heterotrimer or cell type. These differences can govern signaling properties such as adaptation and sensitivity. Compared to a slow translocating βγ complex, a fast translocating βγ complex can help a cell adapt to a signal or protect the cell from the deleterious effects of overactivation of a receptor by rapidly depleting βγ subunits at the plasma membrane. Similarly, in contrast to a fast translocating βγ complex, a slow translocating βγ complex will result in a higher concentration of G protein heterotrimer available for receptor activation at the plasma membrane and lead to higher sensitivity to a signal. Disparate evidence now suggest that differential translocation kinetics does indeed play such roles.

When mammalian cells expressing a slow translocating γ subunit were compared to cells expressing a fast translocating γ subunit for their PLCβ response to increasing doses of a muscarinic receptor agonist, cells with the slow translocating subunit responded at much lower concentrations (111). A similar impact on sensitivity of response was seen when muscarinic receptor activation of GIRK (G protein-coupled inwardly rectifying potassium) channel activity was examined in cardiomyocytes expressing a fast translocating or a slow translocating γ subunit type (91). Cells expressing the slow translocating subunit showed significantly higher current amplitude with same concentration of agonist.

When adrenergic receptor–induced calcium oscillations in human cells was examined, wide variation in the frequency and the duration of oscillations was observed in a population of cells (112). When a fast translocating γ subunit type was expressed in these cells, both the number of spikes and the duration of oscillations decreased significantly among the cells. In contrast, knocking down the same subunit type, γ11 subunit in these cells considerably increased the frequency and oscillation duration compared to control cells. A fast translocating γ subunit thus helps a cell adapt to the external stimulus by dampening the response.

The fast translocating γ1 subunit similarly helps rod photoreceptors adapt to light by translocating away from rhodopsin (113). Adaptation to light is abnormal when γ1 subunit is substituted genetically in mice with a geranylgeranylated mutant that does not translocate efficiently.

When migratory macrophage cells and largely sedentary HeLa cells were compared, the macrophage cells were found to express high levels of slow translocating γ subunits while HeLa cells expressed subunits that translocate relatively faster (96). Macrophage cells showed a strong PIP3 response that was absent in HeLa cells. Introducing a slow translocating subunit, γ3 into HeLa cells resulted in a PIP3 response. Knocking down γ3 or introducing fast translocating γ9 subunit in macrophage cells impaired the PIP3 and the migratory response. Consistent with these findings, another study has shown that the recovery of the PLCβ substrate PIP2 and the cell's ability to adapt to the external stimulus is dependent on the γ subunit constitution of a cell (97). In Gq-mediated PLCβ activation, slow translocating γ3 subunit–sustained effector activity and recovery of substrate was slower, while in the presence of fast translocating γ9 subunit, adaptation was rapid (114). Overall, these studies suggest that a γ subunit type acts as a built-in device which controls a cell's sensitivity and adaptation to signals that activate GPCRs.

To determine how widely such modulation of signaling occurs in cells, studies that focus on quantitatively measuring the translocation, signal input and output simultaneously in real time will be required. Optogenetic methods and live cell imaging are well suited to explore this question further.

Little is known about the potential interaction between α subunits and the translocated βγ complex in internal membranes. Results so far suggest that the translocated βγ complex in internal membranes is free and not bound to an α subunit because it is able to reverse translocate to the plasma membrane as soon as the receptor is deactivated. It would also be of interest to address the following questions. Does free αs.GTP and βγ complex that have translocated to internal membranes act synergistically on the same signaling pathway? Do they form a heterotrimer which is activated by a receptor in internal membranes? Do GPCRs/G proteins in internal membranes modulate the activity of effectors that the translocated βγ complex is regulating?

## Conclusions and perspectives

The normal cellular functions in biological systems such as cardiovascular, nervous, and endocrine are maintained by networks of signaling pathways. Signaling activity is modulated by a series of activation and deactivation events. These events result from intrinsic catalytic activity as in the case of the G protein α subunit and Ras family GTPase switches; posttranslational phosphorylation and dephosphorylation in the case of protein kinases and phosphatases; and second messenger generation in the case of enzymes like adenylyl cyclase and PLCβ or conformational changes as in the case of GPCRs. In contrast, the mechanistic basis of the role that the γ subunits play in modulating signaling is unique. Investigations thus far strongly suggest that the βγ complex–mediated regulation of signaling is primarily determined by the differential affinity that various γ subunit types have for cellular membranes. A small number of residues at the C terminus of the γ subunits determine these differences in affinity and are conserved across species, suggesting that evolutionary pressure was exerted in the case of these proteins on lipid–protein rather than protein–protein interaction.

The potential roles of γ subunits remained relatively less explored because the more obvious functions of α subunits and β subunits attracted considerable attention. Earlier studies that indicated that the γ subunit interacts with a receptor and that this interaction is essential for G protein activation require support from structural data. In the future as methods to capture the transient states of quaternary complexes like receptor–G protein are refined, it is possible that this question will be resolved.

The ability to observe the dynamic properties of signaling proteins in intact living cells has revealed some unexpected roles that this family of small proteins play in signaling. The translocation of βγ complexes allows extracellular signals to act on effectors within the cell. Their differential affinity allows the βγ complex to sustain receptor stimulated effector activity or terminate it rapidly. The γ subunit types can thus determine the sensitivity and intensity of a response as well as adaptation or protection of a cell from an overactivated receptor.

Although G proteins have been studied for decades these roles have begun to emerge only recently. As laboratories with varied technical expertise begin to probe the roles of these subunits, advances are likely to be rapid. Methods such as time-resolved crystallography and solid-state NMR can provide structural information about the C-terminal region of the γ subunit and its potential role in receptor activation of a G protein. Time-resolved crystallography can show how lipidated proteins transition through discrete series of conformations with nanosecond or shorter lifetimes (115). Similarly, cryo-electron microscopy can help determine the three-dimensional structures of proteins at atomic or near-atomic resolution without requiring their crystallization. Deep learning-based methods have been developed to identify the structural movements at the atomic level from these cryo-electron microscopy density maps generated from single particle analysis (116). Application of these methods will soon allow examination of nanosecond movement in distinct domains of lipidated proteins.

To obtain more definitive information about the behavior of G protein subunits in a living cell, live-cell super resolution imaging approaches such as super-resolved structured illumination microscopy (117) and super-resolution radial fluctuations (118, 119), will be helpful. Subcellular optogenetics will continue to be a powerful technique to obtain real time information from the three-dimensional space of a live cell (120). In contrast to ligand-based studies, optical activation and inactivation are almost instantaneous and receptors/G proteins can be activated with an intensity that is precisely controlled unlike diffusible ligands. Light-based activation can be directed at any area in a cell for any period of time to achieve subcellular activation in contrast to complex microfluidic channels. Additionally, subcellular activation of receptors/G proteins is possible with a cell on a surface or in suspension (121). These microscopy techniques together with proteins containing labeled unnatural amino acids or short epitopes such as tetra cysteine motifs, will provide time-resolved distribution changes in G protein subunits at nanometer resolution in living cells.

Knock-in incorporation of an 11-reside peptide HiBiT by using a single-stranded oligo template (122) will allow bioluminescence detection of endogenous G protein subunits. Such tagging will not only have minimal impact on the functional integrity of labeled proteins, but the bioluminescence will also allow protein detection at attomole concentrations. This will be help detect proteins at endogenous expression levels (123).

These methods will help address a number of remaining questions that will help us understand the role of the G protein γ subunits more comprehensively. What are the potential effectors regulated by βγ complexes that translocate to various internal membranes? Does the differential affinity of γ subunit types for membranes play a role in the duration and intensity of signaling at internal membranes? What is the impact of selective expression of γ subunit types on GPCR signaling in various cell types, tissues, and organs? The N-terminal domain of the γ subunits is highly variable within the family but conserved across species (124). Does this diversity among family members and their evolutionary conservation suggest that this domain plays a distinct role?

Receptor-driven βγ complex translocation provides an assay that can be used to screen drugs specific to GPCRs. This assay is useful because all receptor types and all G proteins on activation induce βγ complex translocation. It is reversible so it can be used to identify antagonists which are often the drugs of therapeutic value. The development of optogenetic methods to examine GPCR signaling in live cells was made possible only because of this assay (120, 125, 126). Importantly, apart from optogenetics, this assay can be used for deorphanization and pharmacological drug identification because it can be adapted for high throughput screening (127).

One of the most important areas for future studies is the role of individual γ subtypes in cancer. The evidence thus far for such a role is compelling. Regardless of whether the effects of the misregulated γ subunit types are direct or indirect through a secondary effect on the expression of other components of a signaling pathway, the ability to use the γ subunits as targets for therapy or as markers to predict disease progression are attractive and will need be pursued.

This family of small proteins has attracted less attention and its role largely unnoticed because its functions are dependent on dynamic behavior that can be detected only in living cells which requires fairly sophisticated imaging techniques. Findings so far suggest that future studies may establish a central role for these proteins in normal and pathological signaling.

## Conflict of interest

The authors declare that they have no conflicts of interest with the contents of this article.
